# Efficacy of Metabolically Supported Chemotherapy Combined with Ketogenic Diet, Hyperthermia, and Hyperbaric Oxygen Therapy for Stage IV Triple-Negative Breast Cancer

**DOI:** 10.7759/cureus.1445

**Published:** 2017-07-07

**Authors:** Mehmet Salih İyikesici, Abdul Kadir Slocum, Ayshe Slocum, Ferhan Bulent Berkarda, Miriam Kalamian, Thomas N Seyfried

**Affiliations:** 1 Medical Oncology, Kemerburgaz University Bahcelievler Medical Park Hospital; 2 Medical Oncology, Chemothermia Oncology Center; 3 Nutrition Consultant, Dietary Therapies Llc; 4 Biology, Boston College

**Keywords:** metabolically supported chemotherapy, ketogenic diet, hyperthermia, hyperbaric oxygen therapy, pathological complete response, triple negative breast cancer

## Abstract

Triple-negative breast cancer (TNBC) is more aggressive and metastatic than other breast cancer types. Cytotoxic chemotherapy is presently the predominant systemic therapy for TNBC patients. This case report highlights the influence of metabolically supported chemotherapy (MSCT), ketogenic diet (KD), hyperthermia (HT), and hyperbaric oxygen therapy (HBOT) in an overweight 29-year-old woman with stage IV (T4N3M1) triple-negative invasive ductal carcinoma of the breast. The patient presented with an observable mass in her left breast detected during a physical examination in December 2015. Magnetic resonance imaging revealed a Breast Imaging Reporting and Data System Category 5 tumor and multiple lymphadenomegaly in the left axilla. A Tru-Cut biopsy led to the diagnosis of a triple-negative nuclear grade 2 invasive ductal carcinoma. The patient was admitted to ChemoThermia Oncology Center, Istanbul, Turkey in October 2016, and a whole body (18F)-fluorodeoxyglucose (FDG)-positron emission tomography-computed tomography (PET-CT) scan revealed a 77 mm x 55 mm primary tumor in her left breast, multiple left pectoral and axillary lymph nodes, multiple widespread liver masses, and an upper left nodular abdominal lesion. The patient received a treatment protocol consisting of MSCT, KD, HT, and HBOT. A follow-up whole body 18F-FDG PET-CT scan in February 2017 showed a complete therapeutic response with no evidence of abnormal FDG uptake. The patient continued to receive this treatment protocol and in April 2017 underwent a mastectomy, which revealed a complete pathological response consistent with the response indicated by her PET-CT imaging. This single case study presents evidence of a complete clinical, radiological, and pathological response following a six-month treatment period using a combination of MSCT and a novel metabolic therapy in a patient with stage IV TNBC.

## Introduction

Breast cancer is the most frequently diagnosed cancer among women, with nearly 1.7 million new cases diagnosed worldwide in 2012. It ranks as the fifth cause of death from cancer overall (522,000 deaths) and is the leading cause of cancer death in women [1]. Breast cancer is a heterogeneous disease with several biologically distinct subtypes. Triple-negative breast cancer (TNBC) is defined by the absence of immunohistochemical expression of the estrogen (ER) and progesterone (PgR) receptors and a lack of amplification of the human epidermal growth factor receptor 2 (HER2)/Neu gene, accounting for approximately 20% of breast cancer cases. TNBC has a highly aggressive nature, develops among younger women, and has a higher risk of distant metastasis than other types of breast cancers. Its lack of molecular targets has contributed in part to the difficulty in managing TNBC. Cytotoxic chemotherapy is the only systemic therapy available for these patients. In the treatment of early-stage disease, chemotherapy is effective and pathologic complete response (pCR) rates exceed those of hormonal receptor-positive subtypes [2]. However, patients with metastatic disease experience rapid progression through several lines of chemotherapy. No prior studies have evaluated the influence of metabolically supported chemotherapy (MSCT), ketogenic diet (KD), hyperthermia (HT), and hyperbaric oxygen therapy (HBOT) as a therapeutic strategy for managing TNBC.

The rationale for MSCT is based on Warburg’s hypothesis that “cancer is a disease of metabolic dysregulation” where aerobic fermentation compensates for insufficient oxidative phosphorylation for energy generation [3]. In practice, MSCT initiates with a 12-hour fast, the application of pharmacological doses of regular insulin, and the development of mild hypoglycemia prior to the administration of chemotherapy. As was previously demonstrated in a case report of rectal cancer and a case series in pancreatic cancer, MSCT may enhance the cytotoxic effects of chemotherapy [4-5].

The reduction in circulating glucose can exploit the dependency of cancer cells reliant on glycolytic fermentation. The KD, a high-fat, carbohydrate-restricted diet, decreases blood glucose levels and elevates blood ketone levels, thus slowing the progression of cancer [6]. HT exposes body temperature to 42°C or higher and exploits the heat sensitivity of cancer cells [7]. Tumor hypoxia increases the glycolytic dependency of cancer cells, and hypoxic environments have cancer-promoting effects. HBOT increases oxidative stress specifically in tumor cells and reverses the cancer-promoting effects of hypoxia [8]. We report here a case of stage IV TNBC in a patient that achieved a complete clinical, radiological, and pathological response after receiving a combination of MSCT, KD, HT, and HBOT.

## Case presentation

**Figure 1:**
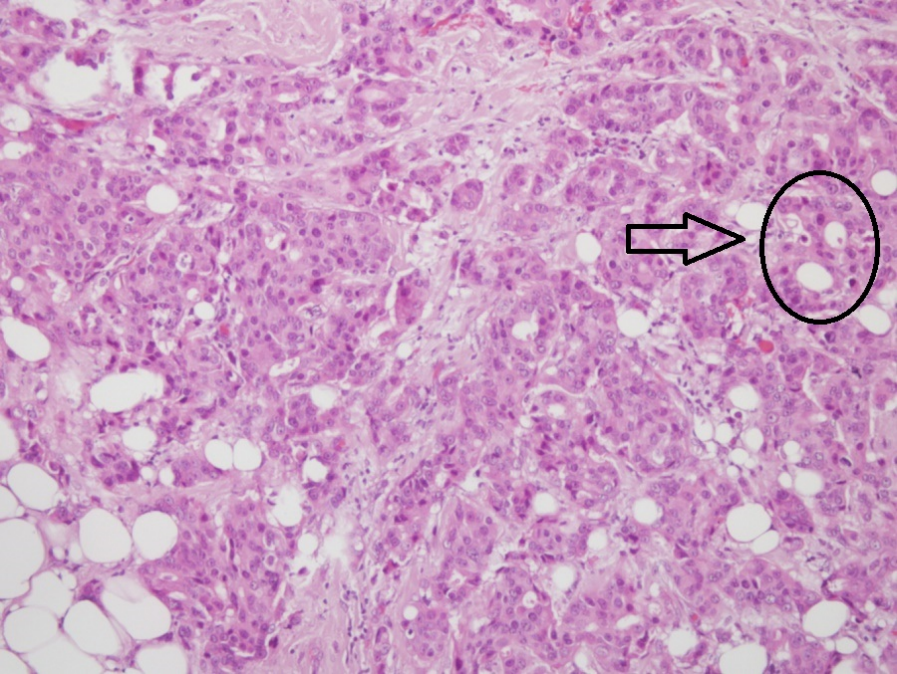
Histopathological examination showing a solid mass and gland forming atypical epithelial cells indicative of nuclear grade 2 invasive ductal carcinoma (x100)

**Figure 2:**
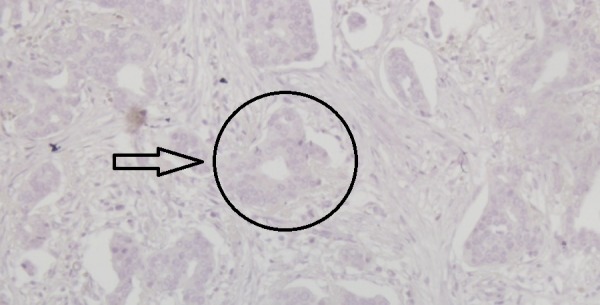
Immunohistochemical examination of the tumor showing negativity for estrogen receptors (x200)

**Figure 3:**
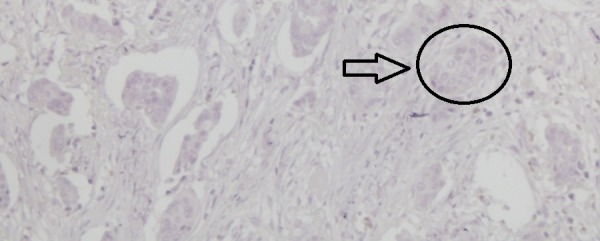
Immunohistochemical examination of the tumor showing negativity for progesterone receptors (x200)

**Figure 4:**
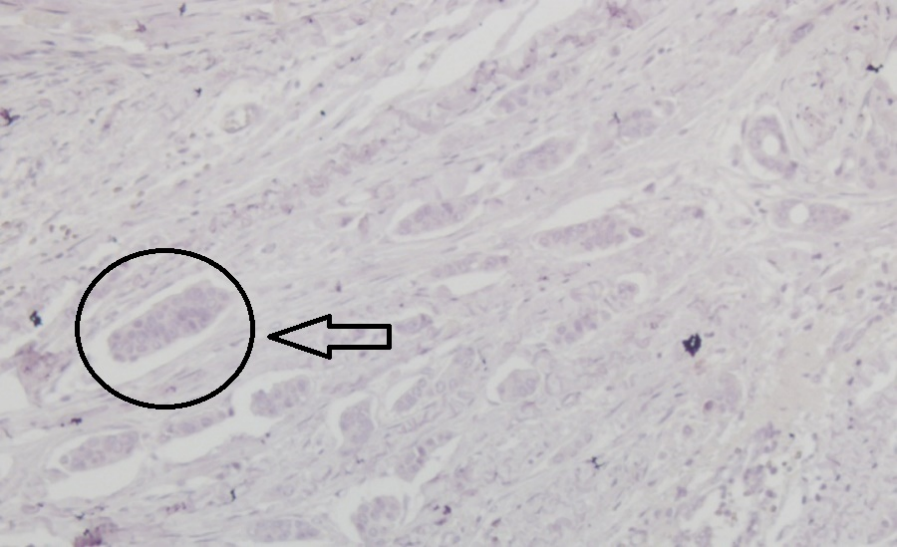
Immunohistochemical examination of the tumor showing negativity for human epidermal growth factor 2 receptors (x200)

An overweight 29-year-old woman with a body mass index (BMI) of 28.1 presented with a lump in her left breast that was detected during a physical examination in December 2015. The patient was admitted to Bakirkoy Dr. Sadi Konuk Education and Research Hospital, Istanbul, Turkey in August 2016, with interval enlargement of the tumor. Magnetic resonance imaging revealed a 75 mm x 75 mm x 65 mm left breast mass (Breast Imaging Reporting and Data System Category 5) with irregular borders. Multiple lymphadenomegaly was seen in the left axilla with the largest being 27 mm x 20 mm. A Tru-Cut biopsy led to a diagnosis of a nuclear grade 2 invasive ductal carcinoma that was negative for ER, PgR, and HER2 receptors (Figures 1-4). 

**Figure 5:**
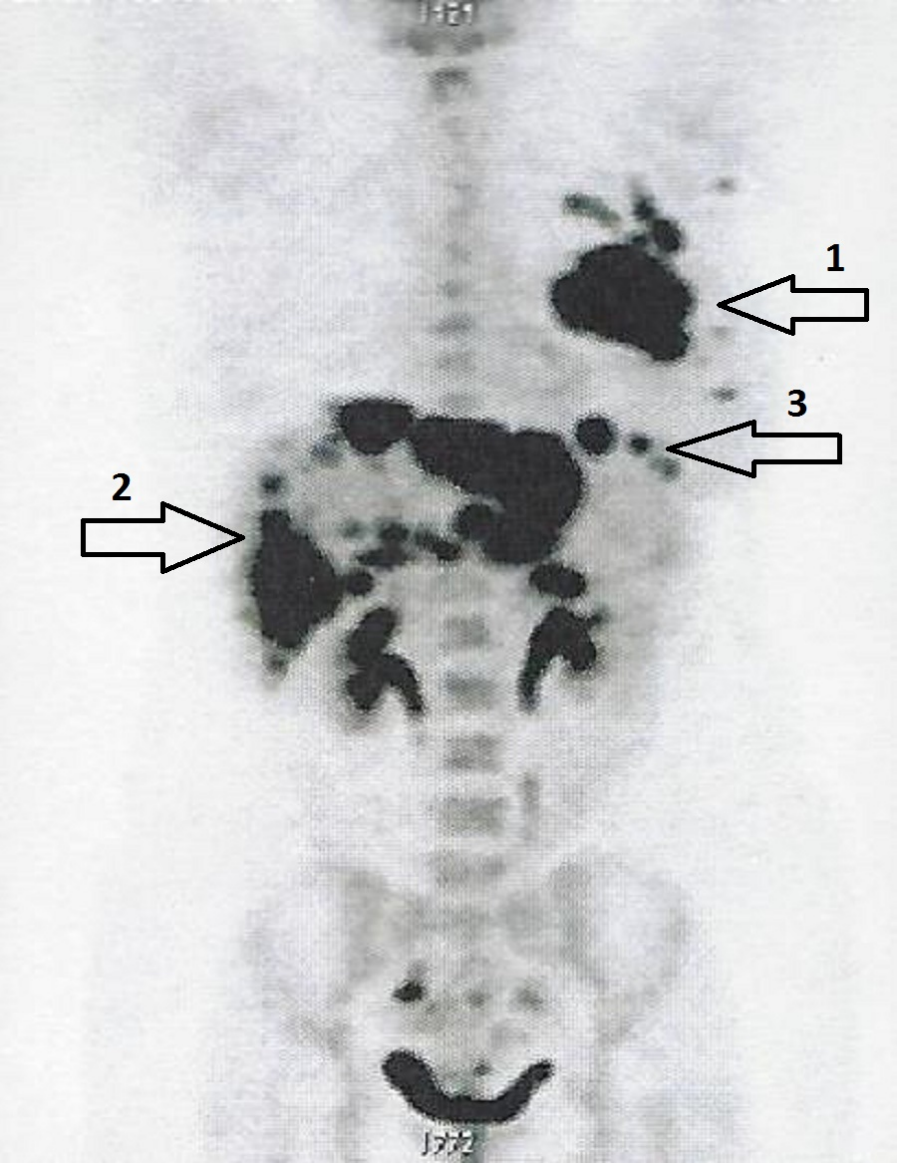
Whole body (18F)-FDG-PET-CT scan showing a 77 mm x 55 mm primary tumor in the left breast (arrow 1), multiple widespread liver masses (arrow 2), and an upper left nodular abdominal lesion (arrow 3). The metastases are so widespread, the use of arrows is nearly insufficient. Abbreviations: CT, computed tomography; FDG, fluorodeoxyglucose; PET, positron emission tomography.

The patient was admitted to ChemoThermia Oncology Center, Istanbul, Turkey on October 1, 2016 and was evaluated using whole body (18F)-fluorodeoxyglucose (FDG)-positron emission tomography-computed tomography (PET-CT). The PET-CT scan revealed a 77 mm x 55 mm primary tumor in her left breast (maximum standard update value [SUVmax]: 22.65), multiple left pectoral and axillary lymph nodes (SUVmax: 11.44), multiple widespread liver masses (SUVmax: 30.34), and an upper left nodular abdominal lesion (SUVmax: 5.94) (Figure 5, Video 1). The patient was diagnosed with stage IV (T4N3M1) triple-negative invasive ductal carcinoma of the breast.

**Video 1:** Coronal fusion video of the whole body (18F)-FDG-PET-CT scan done before treatment on October 1, 2016. Abbreviations: CT, computed tomography; FDG, fluorodeoxyglucose; PET, positron emission tomography.

An MSCT protocol designed for the patient consisted of docetaxel (30 mg/m2), doxorubicin (20 mg/m2), and cyclophosphamide (250 mg/m2). This drug combination was administered following a 12-hour fast and the introduction of 5 to 10 units of regular insulin (Humulin R). Chemotherapy delivery was initiated at blood glucose levels of 50 to 60 mg/dL. With the patient’s written and informed consent, this therapy was delivered on the first and eighth day of a 21-day cycle for a total of four months. Insulin delivery and chemotherapy infusions were delivered after assessing blood glucose levels upon arrival at the clinic, and the insulin dosage was sufficient to lower her blood glucose to approximately 50 mg/dL prior to delivery of the chemotherapy drugs.

In addition to MSCT, the patient was encouraged to consume a KD. She received education regarding the diet restrictions and given food lists as noted in Table 1.

**Table 1:** Ketogenic diet recommendations

Do Eat	Do Not Eat
Eggs	Bread
Leafy Greens	Pasta
Above ground vegetables	Rice
High Fat Dairy	Potatoes
Natural Fats	Sugar
Meats	Honey
Nuts and seeds	Fruits

The patient was not provided with specific recipes or meal plans. Instead, she was directed to modify familiar meals while incorporating more fats. Her blood glucose levels were assessed using a home blood glucose meter (Contour TS, Bayer Health Care, IN, USA). Her urinary ketone levels were also checked prior to each MSCT session and served as a measure of her dietary compliance. The patient remained compliant with the KD presumably because of her understanding of the poor prognosis of this disease and her oncology team’s knowledge about the previously published papers reporting the efficacy and importance of the KD [6,9]. The patient also received local HT and HBOT after each MSCT session. The OncoTherm EHY-3010 HT device (OncoTherm, Troisdorf, Germany) was used to gradually increase her body temperature to 45°C for each hyperthermia session (12 sessions, 60 minutes each) according to the manufacturer’s specifications. A mobile electrode measuring 40 cm x 50 cm was positioned on the thorax and abdomen that fully involved both the primary lesion and the liver metastasis. The Quamvis 320 hyperbaric oxygen chamber (OxyHealth, California, US) was used to produce an operating pressure of 1.5 atmospheres absolute (ATA; 12 sessions, 60 minutes each). The patient tolerated these combined therapies well with no evidence of toxicity or adverse events.

**Figure 6:**
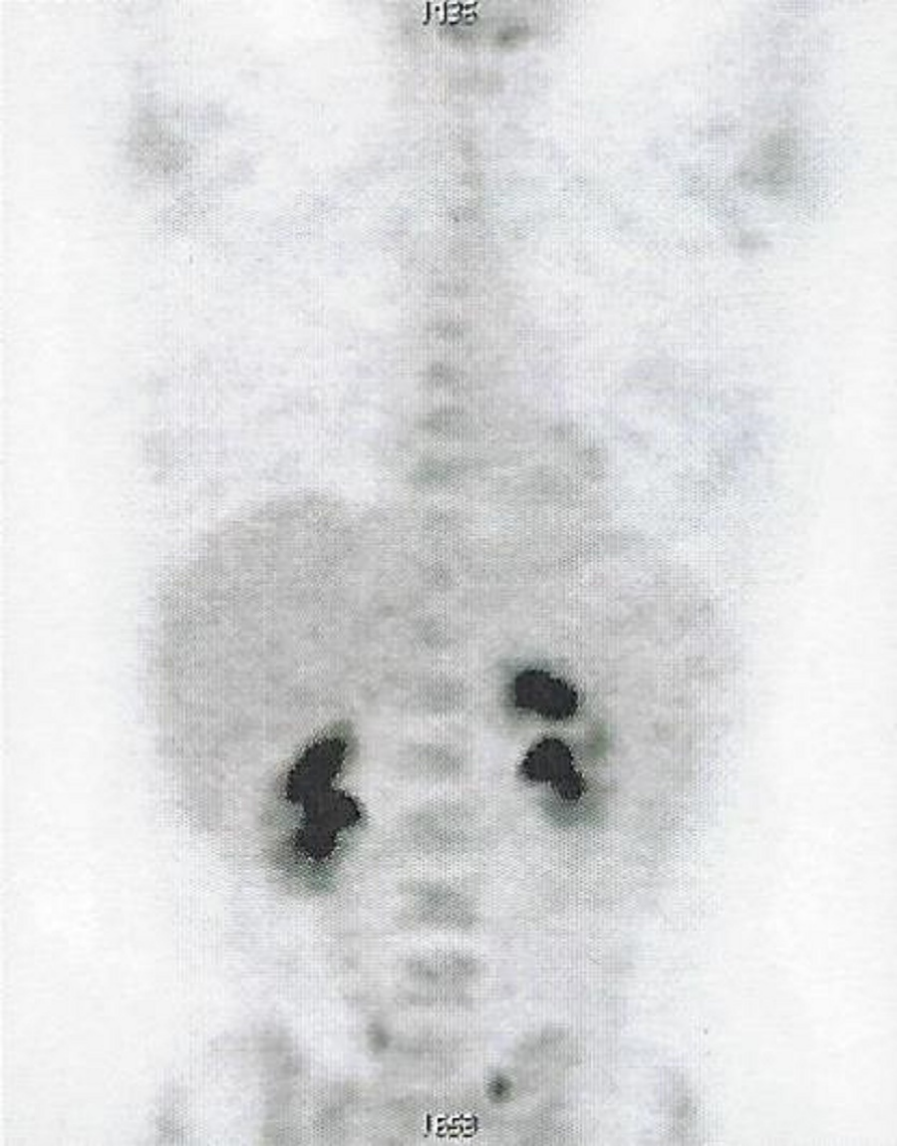
Follow-up whole body (18F)-FDG-PET-CT scan showing no pathological FDG uptake, indicative of a complete response. Abbreviations: CT, computed tomography; FDG, fluorodeoxyglucose; PET, positron emission tomography.

The evaluation of the patient’s whole body (18F)-FDG-PET-CT scan (on February 20, 2017) following the 12 sessions of MSCT, HT, HBOT, as well as KD therapy, demonstrated a complete therapeutic response (Figure 6, Video 2). FDG uptake was no longer detected in any lesions present at the left breast, left axilla, liver, or upper left abdomen.

**Video 2:** Coronal fusion video of the whole body (18F)-FDG-PET-CT scan done following the 12 sessions of MSCT, HT, HBOT, as well as KD therapy on February 20, 2017. Abbreviations: CT, computed tomography; FDG, fluorodeoxyglucose; PET, positron emission tomography.

**Figure 7:**
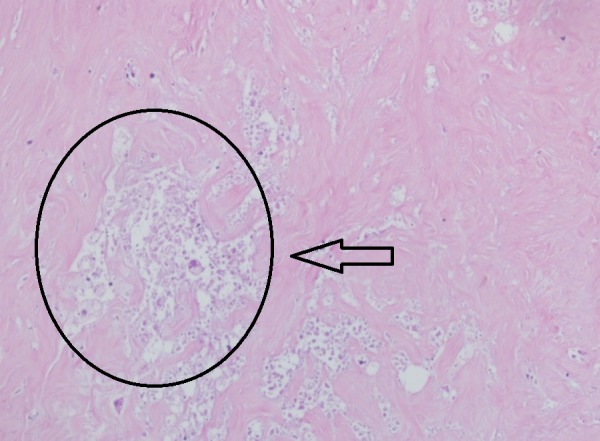
Mastectomy sample of the primary breast tumor area, totally necrotized, showing fibro-hyalinized tissue formed and no live tumor cells; indicative of a pathological complete response (x100)

**Figure 8:**
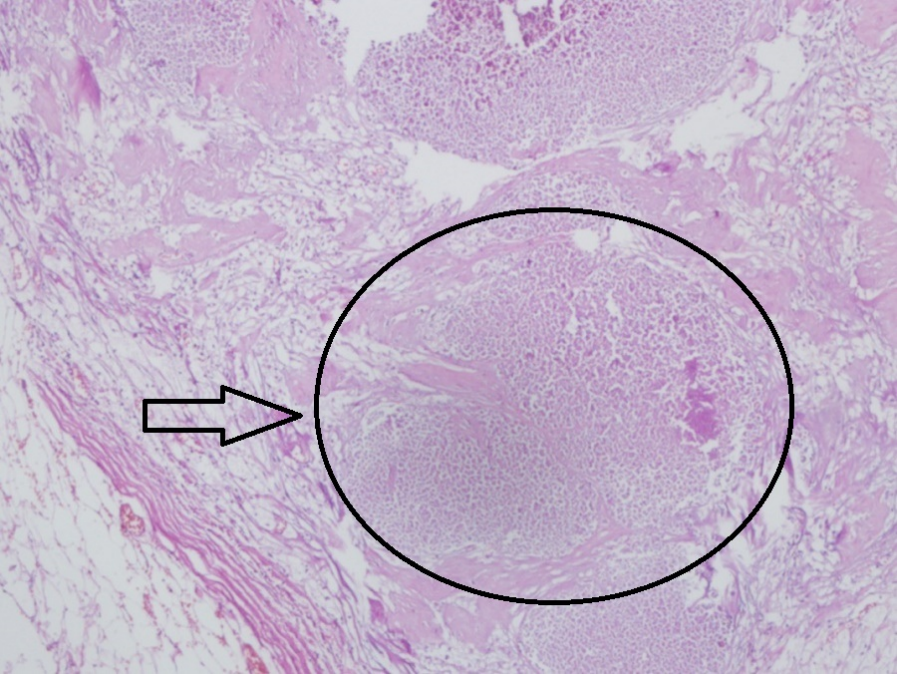
Lymph node sample of the metastatic axillar lymph node showing totally necrotized tissue with no live tumor cells (x100)

The patient’s blood glucose levels averaged 85 mg/dL, and urinary ketones were present at each evaluation (levels reported as + to +++). At the end of the study, the patient’s BMI was 21.8, evidence that she had inadvertently restricted calories, which is known to enhance the metabolic effects of KD therapy [6]. Her self-reported quality of life and energy level had improved significantly compared to the beginning of treatment. The patient continued the same treatment protocol for an additional two months. She then underwent a mastectomy of her left breast with axillary dissection on April 28, 2017. The pathology report identified a 3 cm fibro-hyalinized lesion with no evidence of live tumor cells indicating a pCR consistent with the complete response reported on her PET-CT scan (Figures 7-8). A summary showing the timeline of events is given in Table 2.

**Table 2:** Summary showing timeline of events Abbreviations: CT, computed tomography; FDG, fluorodeoxyglucose; HBOT, hyperbaric oxygen therapy; HT, hyperthermia; KD, ketogenic diet; MRI, magnetic resonance imaging; MSCT, metabolically supported chemotherapy; PET, positron emission tomography; TNBC, triple-negative breast cancer.

Date	Event
December 2015	Lump in left breast detected with physical examination.
December 2015 - August 2016	No further imaging is done.
August 2016	Admitted to Bakirkoy Dr. Sadi Konuk Education and Research Hospital, Istanbul, Turkey where MRI revealed a 75 mm x 75 mm x 65 mm left breast mass with multiple lymphadenomegaly in the left axilla.
August 2016	Tru-cut biopsy confirmed a diagnosis of stage IV TNBC.
October 2016	Admitted to ChemoThermia Oncology Center, Istanbul, Turkey where whole body (18F)-FDG-PET-CT revealed a 77 x 55 mm primary tumor in her left breast together with multiple left pectoral and axillary lymph nodes, multiple wide spread liver masses and an upper left nodular abdominal lesion.
October 2016 - February 2017	Received a treatment protocol consisting of MSCT, KD, HT and HBOT. She received MSCT on the first and eighth day of a 21-day cycle and following each MSCT session she received local HT and HBOT together with being encouraged to consume a KD.
February 2017	Whole body (18F)-FDG-PET-CT demonstrated complete therapeutic response with no malignant FDG uptake following 12 sessions of MSCT, HT, and HBOT together with KD therapy.
February 2017 – April 2017	Continued to receive the same treatment protocol for an additional six sessions.
April 2017	Underwent mastectomy of the left breast with axillary dissection which revealed a pathological complete response.

After achieving this outcome, even though there is no evidence of disease, the presence of microscopic disease cannot be excluded. Consequently, we have decided to continue with the same treatment regimen of MSCT, KD, HT and HBOT to one full year from implementation, taking care not to exceed the cumulative cardiac toxicity dose for doxorubicin, which is part of her MSCT regimen. During this period, the patient will undergo follow-up scans every three months.

## Discussion

We have described a complete response to MSCT, KD, HT, and HBOT in a 29-year-old woman with stage IV (T4N3M1) TNBC that had metastasized to the lymph nodes, liver, and abdomen. There are, currently, no specific treatment guidelines for managing TNBC, and the lack of identifiable molecular targets makes management even more challenging. However, pCR is strongly correlated with a favorable long-term prognosis [10]. We proposed that the effect of standard chemotherapy drugs would be enhanced when combined with therapies that also target the metabolic weaknesses of cancer cells with the goal of achieving pCR. MSCT is a therapeutic strategy that builds on Warburg’s theory that tumor cells lack metabolic flexibility and become dependent on the aerobic fermentation of glucose due to impaired respiration [3-5, 9]. In the case presented here, this therapeutic strategy included the induction of mild hypoglycemia achieved through a 12-hour fast and pharmacological doses of insulin prior to each administration of chemotherapy.

The strong dependence of cancer cells on glucose makes them vulnerable to KDs that lower blood glucose levels while elevating levels of circulating ketone bodies. Ketone bodies are water-soluble energy substrates derived from fatty acid metabolism. They cannot be utilized for energy in cancer cell mitochondria due to respiratory defects [9]. Although the KD has been used for decades as a treatment for intractable pediatric epilepsy, its potential as a therapy for targeting energy metabolism in cancer cells has only recently been explored [6]. The reduced blood glucose levels with elevated urinary ketone levels observed in this patient are theorized to have contributed in part to the patient’s pCR.

In addition to the KD, HT and HBOT also targeted the defective energy metabolism of tumor cells. HT contributes to a therapeutic effect by aiding the uptake of drugs, increasing oxygen radical production, and inhibiting DNA repair in cancer cells, which leads to cancer cell death [7]. HBOT targets tumor hypoxia, which is associated with tumor aggressiveness and resistance to chemotherapy and radiotherapy [8]. Both HT and HBOT also exploit the reliance of tumor cells on glycolysis, a major contributor to the upregulation of antioxidant activity responsible for the tumor’s increased resistance to pro-oxidant chemotherapy and radiation therapies [9]. Consequently, HT and HBOT will selectively increase oxidative stress in the tumor cells. Ketone bodies protect normal cells from this stress while also providing a substrate for energy production. We suggest that the synergistic effect of targeting cancer cell metabolism concurrent with the standard chemotherapy drugs contributed to the patient’s pCR. Moreover, it is important to emphasize that the patient tolerated this treatment well and reported no discomfort or adverse event. This underscores the need to determine if our patient’s response to this treatment was an isolated occurrence or if this response might also be seen in a larger cohort of patients with TNBC.

Despite the advanced stage of this disease, the therapeutic strategy of combining MSCT, KD, HT, and HBOT achieved a clinical and radiological complete response in this patient within four months. The treatment regimen was continued for an additional two months when pCR was further documented in tissue following her mastectomy.

## Conclusions

TNBC is more aggressive and metastatic than other types of breast cancer and has a lack of molecular targets making it more difficult to manage than other cancers. Given the poor prognosis and adverse effects, women with advanced TNBC may be counseled to forego conventional chemotherapy. This single case study presents evidence of a complete clinical, radiological, and pathological response following a six-month treatment period using a combination of MSCT and a novel metabolic therapy in a patient with stage IV TNBC. Given this patient’s remarkable favorable outcomes, further research and randomized clinical trials exploring add-on therapies (such as KD, HT, and HBOT) that may enhance the efficacy of traditional cancer treatments by exploiting the metabolic weaknesses in cancer cells are warranted, especially for patients with poor prognosis of high grade and/or late-stage cancer that is not expected to respond to treatment. Furthermore, this patient did not experience the adverse effects that are commonly associated with the current standard of care and this improved quality of life should also be considered when designing research that compares outcomes of MSCT, KD, HT, and HBOT to traditional treatment. In conclusion, this combined metabolic approach appears effective in treating advanced TNBC, given this patient’s complete response with a good quality of life.
